# pTyr^421^ Cortactin Is Overexpressed in Colon Cancer and Is Dephosphorylated by Curcumin: Involvement of Non-Receptor Type 1 Protein Tyrosine Phosphatase (PTPN1)

**DOI:** 10.1371/journal.pone.0085796

**Published:** 2014-01-22

**Authors:** Vijayababu M. Radhakrishnan, Pawel Kojs, Gavin Young, Rajalakshmy Ramalingam, Bhumasamudram Jagadish, Eugene A. Mash, Jesse D. Martinez, Fayez K. Ghishan, Pawel R. Kiela

**Affiliations:** 1 Department of Pediatrics, Steele Children's Research Center, University of Arizona Health Sciences Center, Tucson, Arizona, United States of America; 2 Department of Nutritional Sciences, Tucson, Arizona, United States of America; 3 Arizona Cancer Center, Tucson, Arizona, United States of America; 4 Department of Immunobiology, University of Arizona Health Sciences Center, Tucson, Arizona, United States of America; 5 Department of Chemistry and Biochemistry, The University of Arizona, Tucson, Arizona, United States of America; Wayne State University School of Medicine, United States of America

## Abstract

Cortactin (CTTN), first identified as a major substrate of the Src tyrosine kinase, actively participates in branching F-actin assembly and in cell motility and invasion. *CTTN* gene is amplified and its protein is overexpressed in several types of cancer. The phosphorylated form of cortactin (pTyr^421^) is required for cancer cell motility and invasion. In this study, we demonstrate that a majority of the tested primary colorectal tumor specimens show greatly enhanced expression of pTyr^421^-CTTN, but no change at the mRNA level as compared to healthy subjects, thus suggesting post-translational activation rather than gene amplification in these tumors. Curcumin (diferulolylmethane), a natural compound with promising chemopreventive and chemosensitizing effects, reduced the indirect association of cortactin with the plasma membrane protein fraction in colon adenocarcinoma cells as measured by surface biotinylation, mass spectrometry, and Western blotting. Curcumin significantly decreased the pTyr^421^-CTTN in HCT116 cells and SW480 cells, but was ineffective in HT-29 cells. Curcumin physically interacted with PTPN1 tyrosine phosphatases to increase its activity and lead to dephosphorylation of pTyr^421^-CTTN. PTPN1 inhibition eliminated the effects of curcumin on pTyr^421^-CTTN. Transduction with adenovirally-encoded CTTN increased migration of HCT116, SW480, and HT-29. Curcumin decreased migration of HCT116 and SW480 cells which highly express PTPN1, but not of HT-29 cells with significantly reduced endogenous expression of PTPN1. Curcumin significantly reduced the physical interaction of CTTN and pTyr^421^-CTTN with p120 catenin (CTNND1). Collectively, these data suggest that curcumin is an activator of PTPN1 and can reduce cell motility in colon cancer via dephosphorylation of pTyr^421^-CTTN which could be exploited for novel therapeutic approaches in colon cancer therapy based on tumor pTyr^421^-CTTN expression.

## Introduction

Cortactin, encoded by the *CTTN/EMS1* gene, is a v-Src substrate localized with cortical actin at the plasma membrane and is overexpressed in many types of cancer [1]. Cortactin overexpression results from the 11q13.3 chromosomal region amplification in various cancers, such as head and neck squamous carcinoma, hepatocellular carcinoma, breast and bladder cancer, and correlates with poor patient prognosis and decreased survival [2]–[5]. Cortactin, generally present in several different cell types, is enriched in cortical structures such as membrane ruffles and lamellipodia, and plays key roles in the microfilament-membrane interactions as well in transducing signals from the cell surface to the cytoskeleton [6], [7]. Cortactin actively participates in Arp2/3-mediated actin polymerization associated with the plasma membrane [7] and acts as an F-actin modulator in tyrosine kinase-regulated cytoskeleton reorganization [8] suggesting a mechanism for its role in motility. Its role in cell migration and invasion is well studied in epithelial cells, fibroblasts, endothelial cells, and breast cancer cells [8]–[10]. Phosphorylation of murine cortactin at Tyr^421^, Tyr^466^ (Tyr^470^ in humans) and Tyr^482^ (Tyr^486^ in humans) is required for efficient cell motility in several cell types, indicating that cortactin tyrosine phosphorylation plays an important role in cell migration [8], [11], [12]. Generally, tyrosine phosphorylation of cortactin triggers recruitment of SH2-domain proteins, including several kinases and the NCK adaptor protein NCK1, which links cortactin with Wiskott-Aldrich syndrome-like protein (WASL, N-WASP) and WAS/WASL interacting protein family member 1 (WIF1, WIP). This in turn leads to enhanced activation of the Arp2/3 complex (actin-related protein 2 homolog/3 homolog) and leads to actin filament branching [13]–[16].

Numerous epidemiological studies have shown that plant based phenolic compounds in dietary agents play important roles in chemoprevention of colorectal cancer [17], the second most common cancer in men and third most common in women. Regular consumption of fruits and vegetables containing these compounds has been associated with a decreased incidence of colorectal cancer [18]. Among the natural bi-phenolic compounds, curcumin, a curcuminoid from the rhizome *curcuma longa*, is well known for its anti-cancer, and anti-inflammatory, antioxidant activity in vivo and in vitro [19]–[21], and is well-tolerated in large doses. In a phase II study with advanced pancreatic cancer patients, a dose of 8 g was administered for 2 months with observed toxicity [22]. Another phase I clinical trial evaluated the tolerability of curcumin in 25 subjects with high risk precancerous lesions. Histological improvements were observed in 7 of the 25 subjects, with no treatment-related toxicity up to 8 g/day [23]. The anticancer effects of curcumin and its derivatives have typically been attributed to inhibition of cell proliferation, cell cycle arrest, and/or induction of apoptosis [21], [24], [25]. One clinical study showed that administration of doses of up to 2.2 g of *Curcuma* extract (containing 180 mg of curcumin) per day for up to 4 months showed clinical benefits in patients with advanced refractory colorectal cancer [26].

In the present study, we demonstrate that pTyr^421^ cortactin is overexpressed in colorectal cancer without concomitant changes in mRNA levels. Curcumin decreased the levels of pTyr^421^ cortactin in colon cancer cells *in vitro* by physically interacting with the non-receptor type 1 protein tyrosine phosphatase (PTPN1; PTP1b) to increase its activity, and dephosphorylate cortactin, thus reducing cancer cell migration. Our data suggest potential usefulness of pTyr^421^ cortactin immunostaining as a biomarker of invasive colon cancer and provide further insight into the mechanism for chemopreventive effects of curcumin and its potential role in preventing metastatic colon cancer.

## Materials and Methods

### Reagents

Curcumin with 98.05% purity and free of contaminating curcuminoids (demethoxy-curcumin and bis-demethoxy curcumin), was custom-purified by ChromaDex (Irvine, CA). PTPN1 inhibitor XXII (3-(3,5-dibromo-4-hydroxy-benzoyl)-2-ethyl-benzofuran-6-sulfonicacid-(4-(thiazol-2-ylsulfamyl)-phenyl)-amide), a cell-permeable, selective, reversible, and a non-competitive allosteric inhibitor of PTPN1 [27] was obtained from EMD Millipore (Billerica, MA). Recombinant adenoviral cortactin was obtained from Vector Biolabs (Philadelphia, PA).

### Antibodies, cell lines and human tissues

T-84 cells (human colorectal carcinoma) originally described by Murakami and Masui [28] were provided by Dr. Declan McCole, University of California San Diego, CA. HCT116, HT29 and SW480 cells were obtained from ATCC and were cultured in Dulbecco's Modified Eagle Medium (DMEM; Gemini Bio Products, West Sacramento, CA), 10% Fetal Bovine Serum (Cellgro, Manassas, VA), and 1% Penicillin-Streptomycin (Life Technologies, Grand Island, NY). Cells were grown in a 10 cm dish or in six well plates (Greiner Bio-One, Monroe, NC). Mouse monoclonal anti-GAPDH, rabbit polyclonal phospho-specific (pTyr^421^) cortactin antibody, and anti-PTPN1 antibody were purchased from EMD Millipore. Anti–cortactin mouse monoclonal and anti-cortactin rabbit polyclonal antibodies were obtained from Santa Cruz Biotechnologies, (Santa Cruz, CA). Mouse monoclonal anti-p120 catenin antibody was purchased from BD Biosciences, (San Jose, CA). Frozen human colon tumor specimens and non-malignant tissues were obtained from Cooperative Human Tissue Network, Vanderbilt University Medical Center (Nashville, TN). 44 samples were selected based on the tumor type and percentage of tumor cell content (>80%) along with 37 normal tissues. These studies were evaluated by the University of Arizona Human Subjects Protection Program and judged to be exempt as the specimens were de-identified.

### qRT-PCR

Total RNA was extracted from the tissues using TRIZOL according to the manufacturer's protocol (Life Sciences). 500 ng of total RNA was reverse-transcribed by using a cDNA synthesis kit (Bio-Rad; Hercules, CA). The qPCR was performed with Taqman qPCR mix (Quanta BioSciences; Gaithersburg, MD) and predesigned TaqMan primers and probes (all from Applied Biosystems/Life Technologies) according to the manufacturer's protocol with iCycler CFX96 (Bio-Rad). The qPCR measurement was assessed based on the relative quantification of CTTN gene normalized to the expression of GAPDH. The data were expressed as ΔCt values and the Student's t test was used to analyze the data.

### Immunohistochemistry

Colorectal cancer tissue array of paraffin embedded sample cores was obtained from US BioMax Inc (Rockville, MD). Phosphospecific anti-pTyr^421^ cortactin and total cortactin antibodies were used for immunostaining. The slides were deparaffinized, rinsed with Phosphate Buffered Saline (PBS; pH 7.4) and blocked with goat serum (Santa Cruz). They were then incubated with the primary antibodies for 4 h at 4°C. The slides were washed and then incubated with the goat anti-rabbit HRP-conjugated antibody (Santa Cruz) at room temperature for 1 h. The slides were developed with a DAB substrate kit (Vector Labs) and counterstained with hematoxylin.

### Cell surface biotinylation

Apical cell surface biotinylation was performed as described previously [29]. Briefly, 2×10^5^ T84 colon cancer cells were seeded on Transwell filters (Corning Incorporated) and grown for 5 –7 days until the monolayer resistance reached at least 600 Ω^.^cm^2^. Cells were then treated with curcumin (50 µM) or dimethyl sulfoxide (DMSO, vehicle) at the apical side for 1 hour. Surface proteins were biotinylated by applying 0.5 ml of NHS-S-S-biotin (Pierce; Rockford, IL) in PBS for 30 min at 4°C at the apical side. After quenching, filters were excised and cells were lysed with 0.5 ml RIPA lysis buffer containing protease and phosphatase inhibitors (Halt protease/phosphatase inhibitor cocktail; Pierce). Lysed samples were briefly sonicated, centrifuged at 13,000 rpm for 10 min at 4°C, and the supernatant was collected and used for protein assay and pull-down experiments. Biotinylated proteins were pulled down with streptavidin-agarose beads (Pierce). Bound proteins were eluted by incubating the beads in radioimmunoprecipitation assay buffer (RIPA; 50 mM Tris HCl pH 7.42, 150 mM NaCl, 1% NP-40, 0.25% Na-deoxycholate, protease and phosphatase inhibitor cocktails, and phenylmethylsulfonyl fluoride) at 98°C for 5 min. The samples were centrifuged at 10,000 rpm for 5 minutes, and the supernatant was separated and mixed with 0.125 ml of trichloroacetic acid (TCA), incubated for 10 min on ice, and centrifuged at 14,000 rpm for 5 min. Supernatants were discarded and pellets washed three times with ice-cold acetone. The pellets were air-dried and protein quantified before it was processed for MS analysis. For immunoblotting, proteins were separated by SDS-PAGE, followed by immunoblotting with anti-cortactin antibody. Even loading of biotinylated surface proteins was confirmed with goat polyclonal anti-transferrin receptor antibody (Santa Cruz).

### Protein identification by quadrupole time of flight tandem mass spectrometry (QTOF MS/MS)

Four hundred nano grams of cell membrane-associated proteins, obtained as described above, were processed for tryptic digestion and analysed by liquid chromatography-mass spectrometry by the Arizona Proteomics Consortium shared facility. QTOF MS/MS analysis of trypsin digested proteins were carried out using a quadrupole time-of-flight mass spectrometer (QTOF; Waters Q-TOF Premier, 2008). Peptides were eluted using Vydac C18 (Hesperia, CA), using a gradient of 0–65% solvent (98% methanol/2% water/0.5% formic acid/0.01% triflouroacetic acid) over a 60-min period at a flow rate of 350 nl/min. The peptide solution was sprayed at a potential of 1.6 kV, and the capillary temperature at 200°C. Dependent data scanning was performed by the Xcalibur v 2.0 SR2 software [30] with a default charge of 2, an isolation width of 1.5 amu, an activation amplitude of 35%, activation time of 30 msec, and a minimal signal of 10,000 ion counts. Global dependent data settings were as follows: reject mass width of 1.5 amu, dynamic exclusion enabled, repeat count of 1, repeat duration of 1 min, and exclusion duration of 5 min. Scan event series included one full scan with mass range 350 – 2000 Da, followed by 3 dependent MS/MS scans of the most intense ion. Dynamic exclusion was turned on for a period of 60 sec. Tandem MS spectra of peptides were analyzed with Turbo SEQUEST™ v 3.1, a program that allows the correlation of experimental tandem MS data with theoretical spectra generated from known protein sequences. The search criteria that were used for a preliminary positive peptide identification are the same as previously described, namely peptide precursor ions with a +1 charge having a Xcorr >1.8, +2 Xcorr > 2.5 and +3 Xcorr > 3.5. A dCn score > 0.08 and a fragment ion ratio of experimental/theorical >50% were also used as filtering criteria for reliable matched peptide identification [31]. All matched peptides were confirmed by visual examination of the spectra. All spectra were searched against the ipiHuman 3.72 protein database which has therein the sequence of the *Rhodobacter* and bovine serum albumin. Usually, bovine serum albumin is added as a reference protein but since bovine and human serum albumin are similar, we used the T33V mutant sequence of *Rhodobacter*. Scaffold (version Scaffold_3.1.2, Proteome Software Inc., Portland, OR) was used to validate MS/MS based peptide and protein identifications. Peptide identifications were accepted if they could be established at greater than 90.0% probability as specified by the Peptide Prophet algorithm [30]. Protein identifications were accepted if they could be established at greater than 90.0% probability and contained at least one identified peptide. Protein probabilities were assigned by the Protein Prophet algorithm [32]. Proteins that contained similar peptides and could not be differentiated based on MS/MS analysis alone were grouped to satisfy the principles of parsimony.

### Western blotting and immunoprecipitation

The protein lysates obtained from colon tumor tissues and colon cancer cell lines were prepared with RIPA buffer. The precleared protein samples were quantified [DC (Detergent-compatible) colorimetric protein assay kit; Bio-Rad], and samples were separated by SDS-PAGE followed by immunoblotting. The blots were visualized with SuperSignal West Pico Chemiluminescent Substrate (Pierce). For immunoprecipitation, cells were lysed with RIPA Buffer and protein was quantified with a DC protein assay kit (Bio-Rad). 5 µg of anti-pTyr^421^ cortactin antibody was added to 1 mg of HCT-116 cell lysates at 4°C and incubated overnight. The samples were then incubated with A/G protein agarose beads (Santa Cruz Biotechnology, Santa Cruz, CA) for 1 h at 4°C. Immunoblotting was performed from the washed and denatured complexes.

### Immunofluorescence

Cells were grown in EZ-chambers (Millipore), and treated with curcumin at 50 µM concentration for 15 min and were fixed with 2% paraformaldehyde in 0.2 M phosphate buffer, pH 7.4 (45 min), permeabilized with 0.1% Triton X-100 in PBS (10 min), and incubated sequentially with anti-phospho cortactin (Millipore) or anti-cortactin antibody (Santa Cruz). The Alexa Fluor 647 goat anti-rabbit IgG, (Life Technologies) was used as secondary antibody. The slides were mounted using fluorescence mounting medium (Dako; Carpinteria, CA) and were examined with a Zeiss confocal microscope equipped with ZEN software (Carl Zeiss Microscopy, GmbH).

### PTPB1B/PTPN1 activity assay

HCT 116 cells were grown until confluence and treated with curcumin or DMSO (vehicle) in media for 30 min. Cells were washed with PBS and lysed with RIPA Buffer, sonicated for 5 seconds, centrifuged, and assayed for protein concentration. A PTPB1B Assay kit was obtained from Millipore and used according to the manufacturer's instructions.

### Sequencing of PTPN1 coding region

Total RNA was extracted from HCT116, HT29, and SW480 cells using TRIZOL (Life Technologies) according to the manufacturer's instructions, reverse transcribed using a cDNA synthesis kit (Quanta), and amplified using forward primer, 5′-ATGGAGATGGAAAAGGAGTTCG-3′ and reverse primer, 5′-CTATGTGTTGCTGTTGAACAGGA-3′ (based on Gene Accession NM_002827.2) and *Pfu* Taq DNA polymerase (Life Technologies). PCR products were gel purified, subcloned into pGEM-T Easy (Promega, CA), and sequenced from 5′ and 3′ ends using T7 and SP6 sequencing primers.

### Cell migration assay

The transwell migration assay was performed as reported earlier [33] with minor modifications. Briefly, Transwell membrane (24-well insert, pore size 8 µm, polycarbonate; Corning Inc, NY) was used to determine the effect of curcumin on colon cancer cell migration *in vitro*. The cells were trypsinized, washed, and kept suspended in medium without FBS. To the lower wells of the chambers, migration-inducing medium (with 10% FBS) was added. Upper wells were filled with serum-free medium with cells (20,000 cells per well), in some cases, also containing 25 µM of curcumin. Then, the chamber was placed into a humidified CO_2_ incubator. After 12 h, assays were stopped by removal of the medium from the upper wells and careful removal of the filters. Filters were fixed with methanol by brief submersion and cells from upper side were removed by washing with PBS. Filters were stained with crystal violet staining (0.2%, w/v with ethanol 2%, v/v, in 0.5M Tris-HCl, pH 7.8) for 10 min at room temperature. The stained cell layer was rinsed thoroughly with 0.5 M Tris-HCl (pH 7.8) three times, filters were air-dried, incubated with 500 µl SDS solution (0.5% in 50% ethanol, 50% 0.5 M Tris-HCl, pH 7.8) for 60 min at 37°C, and read at 586 nm using a spectrophotometer (Molecular Devices, CA). For Ad-CTTN transduced cells, 24 h following infection with Ad-CTTN (10 MOI), cells were trypsinized, washed with PBS, and added to the upper side of the Transwell chamber, and assays were performed as described above.

### Chemical synthesis of biotinylated curcumin

Reactions were conducted using flame-dried glassware under a positive pressure of argon. Hydroscopic solvents were transferred *via* an oven-dried syringe or cannula. All reagents and solvents were commercially available and were used as received. Solutions were concentrated *in vacuo* using a rotary evaporator. Analytical thin-layer chromatography (TLC) was performed on pre-coated silica gel 60 F-254 glass plates. TLC visualization required using an iodine chamber, UV light, and/or PMA solution (5 g phosphomolybdic acid, 100 mL 95% EtOH) for staining. Flash and gravity chromatography were performed using silica gel 60 (230–400 mesh). Melting points are uncorrected. Nuclear Magnetic Resonance (NMR) experiments were performed on a 500 MHz spectrometer. NMR spectra were referenced to CD_3_OD (3.31 ppm, 49.0 ppm). Mass spectrometry was conducted using ESI on a Bruker Daltonics MALDI TOF instrument.

#### N-(13-Amino-4,7,10-trioxatridecanyl)biotinamide (2)

To a warm solution of biotin (0.24 g, 1.0 mmol) and *N*-hydroxysuccinamide (0.12 g, 1.0 mmol) in DMF (8 mL) was added DCC (0.26 g, 1.3 mmol) and the reaction mixture stirred overnight at room temperature. The solids were removed by filtration, washed with DMF (2 mL), and the filtrate (∼10 mL) added dropwise to a stirred solution of 4,7,10-trioxa-1,13-tridecanediamine (2.20 g, 10.0 mmol, 2.2 mL) in DMF (20 mL). After 3 h, the solvent was removed under reduced pressure. The resulting oil was triturated with ether (50 mL) and the mixture was stirred for 30 min. The solid was collected by filtration and subjected to flash column chromatography on silica gel (50 g). Elution with methanol/EtOAc (4∶1) gave **2** (0.38 g, 0.85 mmol, 85% over two steps) as a colorless solid, mp 104–106°C, R*_f_* 0.36 (MeOH/EtOAc/aq. NH_4_OH 8∶2∶1). The ^1^H and ^13^C NMR spectra were in agreement with published data [34].

#### 5,21-Dioxo-25-((3aS,4S,6aR)-2-oxohexahydro-1H-thieno[3,4-d]imidazol-4-yl)-10,13,16-trioxa-6,20-diazapentacosan-1-oic Acid (3)

To a solution of **2** (0.38 g, 0.85 mmol) in methanol (4 mL) was added glutaric anhydride (0.12 g, 1.02 mmol) and the mixture stirred overnight. Solvent was removed under reduced pressure and the residue subjected to flash column chromatography on silica gel (50 g). Elution with CH_2_Cl_2_ (200 mL) followed by CH_2_Cl_2_/MeOH (5∶1) gave **3** (0.40 g, 0.71 mmol, 84%) as a white solid, mp 124–126°C, R*_f_* 0.42 (CH_2_Cl_2_/MeOH 1∶1). ^1^H NMR (500 MHz, CD_3_OD) δ 1.44 (2, m), 1.56–1.70 (4, m), 1.70–1.78 (6, m), 1.88 (2, quintet, *J* = 7.5 Hz), 2.18–2.25 (4, m), 2.32 (2, m), 2.70 (2, m), 2.93 (2, dd, *J* = 8 Hz, 5 Hz), 3.19–3.21 (2, m), 3.25 (4, t, *J* = 7 Hz), 3.52 (4, m), 3.59 (5, m), 3.64 (5, m), 4.30 (1, m), 4.49 (1, m); ^13^C NMR (125 MHz, CD_3_OD) δ 22.3, 26.8, 29.5, 29.7, 30.4, 34.2, 36.1, 36.8, 37.8, 41.0, 56.9, 61.26, 63.3, 69.9 (2), 71.2, 71.5, 166.1, 175.2, 175.9, 176.8; HRMS (MALDI TOF) calculated for C_25_H_45_N_4_O_8_S 561.2952, observed 561.2930.

#### 4-((1E,6E)-7-(4-Hydroxy-3-methoxyphenyl)-3,5-dioxohepata-1,6-dien-1-yl)-2-methoxyphenyl-5,21-dioxo-25-((3aS,4S,6aR)-2-oxohexahydro-1H-thieno[3,4-d]imidazol-10,13,16-trioxa-6,20-diazapentacosan-1-oate (4)

To a solution of **3** (100 mg, 0.18 mmol) in DMF (2 mL) was added *N*-hydroxysuccinamide (21 mg, 0.18 mmol) followed by DCC (48 mg, 0.23 mmol) and the reaction mixture stirred overnight at room temperature. Solids were removed by filtration, washed with DMF (2 mL), and the filtrate (∼4 mL) added dropwise to a stirred solution of curcumin (330 mg, 0.89 mmol) and triethylamine (45 mg, 0.44 mmol, 62 µL) in DMF (10 mL). The reaction mixture was stirred overnight at room temperature and volatiles removed under reduced pressure. The residue was subjected to flash column chromatography on silica gel (75 g). Elution with CH_2_Cl_2_ (200 mL) and CH_2_Cl_2_/MeOH (50∶1, 200 mL) gave curcumin. Further elution with CH_2_Cl_2_/MeOH (20∶1), followed by removal of solvents, gave **4** as a dark yellow gum which was dissolved in 20% acetontrile in water and the solution freeze-dried. Biotinylated curcumin derivative **4** (78 mg, 0.085 mmol, 48% yield over the two steps) was obtained as a fluffy dark yellow solid, R*_f_* 0.68 (CH_2_Cl_2_/MeOH 5∶1). ESI-MS: 911.2 (M+H^+^). The ^1^H NMR spectrum was in agreement with published data [35].

### Statistical analysis

Unpaired Student's t-test was utilized for statistical analysis throughout.

## Results

### pTyr^421^cortactin expression is increased in primary colon cancer tissues

**Figure 1:**
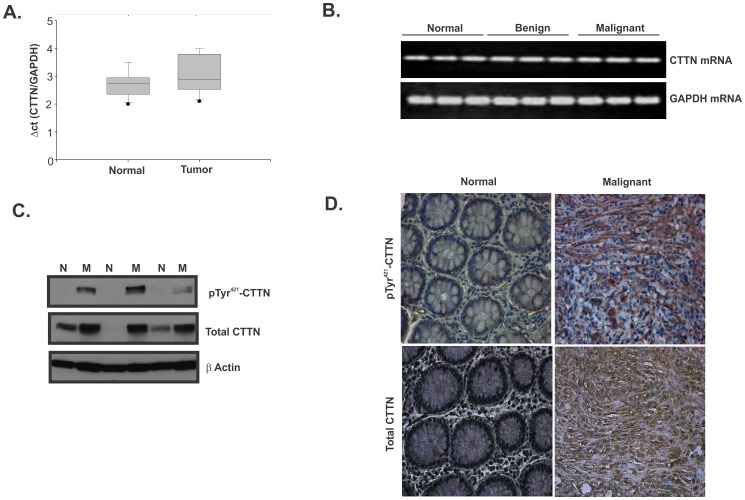
Expression of cortactin in human colon adenocarcinomas samples. (**A**) Total RNA was extracted from frozen samples of normal and tumor tissues and cortactin mRNA expression was determined using quantitative RT-PCR. The box plots depict relative quantities of cortactin normalized to GAPDH in normal and tumor tissues (n = 37 for normal samples, n = 44 for tumor samples). (**B**) DNA gel analysis of PCR products obtained from qPCR analysis (representative of 37 normal tissues, 5 benign tumors, and 39 malignant tumors). (**C**) The tissue lysates of matched pairs (N-normal, M-malignant) of colon specimens prepared from the same tumor samples as in (A) were analyzed the expression of pTyr^421^-cortactin (pTyr^421^-CTTN) and total cortactin by Western blotting. Representative results are shown from three matched pairs. β-actin was used as a loading control. (**D**) Representative pTyr^421^-cortactin and total cortactin immunostaining of colon tumor specimens. Tissue array containing a series of colon carcinomas were stained for pTyr^421^-CTTN and total cortactin. The staining was mostly cytoplasmic for total cortactin, whereas pTyr^421^-CTTN showing increased staining intensity at the plasma membrane.

Overexpression of cortactin has been found in invasive cancers, including glioblastoma, melanoma, breast cancer, and head and neck squamous carcinomas, as a result of the EMS1 gene amplification [36]. In this study, we investigated cortactin expression in colon cancer. We first analyzed cortactin mRNA expression in 81 frozen colon tissue samples, which included 37 normal tissues, 5 benign, and 39 malignant tumors, by quantitative RT-PCR. Colon tumors and adjacent normal tissues showed no difference in cortactin mRNA expression (Fig 1A). We observed no significant difference between CTTN mRNA expression in normal tissues and colon tumor samples (Fig 1B). Next, we examined 19 matched pairs of colon tumor specimens for cortactin protein expression by western blot. 14/19 (73%) showed elevated levels of pTyr^421^ cortactin and total cortactin (Fig. 1C, left panel), 2/19 samples showed decreased expression of both forms (pTyr^421^ and total) of cortactin, and 3/19 showed no change (data not shown). Immunohistochemical analysis of tissue arrays with 6 normal sections and 18 colon tumor sections showed intense membrane staining of pTyr^421^ cortactin and total cortactin in 13/18 tumor samples analyzed as compared to normal tissue sections, as exemplified in Fig. 1D. We also investigated cortactin phosphorylation at Tyr^482^ and Tyr^470^ in colon tumor samples. We found that in 50% of the analyzed malignant tissues, there was an increased expression of pTyr^482^ when compared with matched controls (data not shown). However, phosphorylation at this site was not affected by curcumin in HCT116 cells (not shown), and since Oser et al [37]. demonstrated that pTyr^482^ does not contribute to the regulation of the number of free barbed ends in invadopodia, we did not pursue this site further. We were unable to reliably analyze the expression of pTyr^470^ in tumor specimens with any commercially available antibodies.

Discrepancy between unaltered mRNA expression and elevated levels of total and phosphorylated cortactin excluded the possibility of gene amplification and suggested possible posttranslational modification(s) and changes in protein stability in colorectal tumors.

### Changes in cell surface biotinylated proteins after curcumin treatment in T84 cells

**Figure 2:**
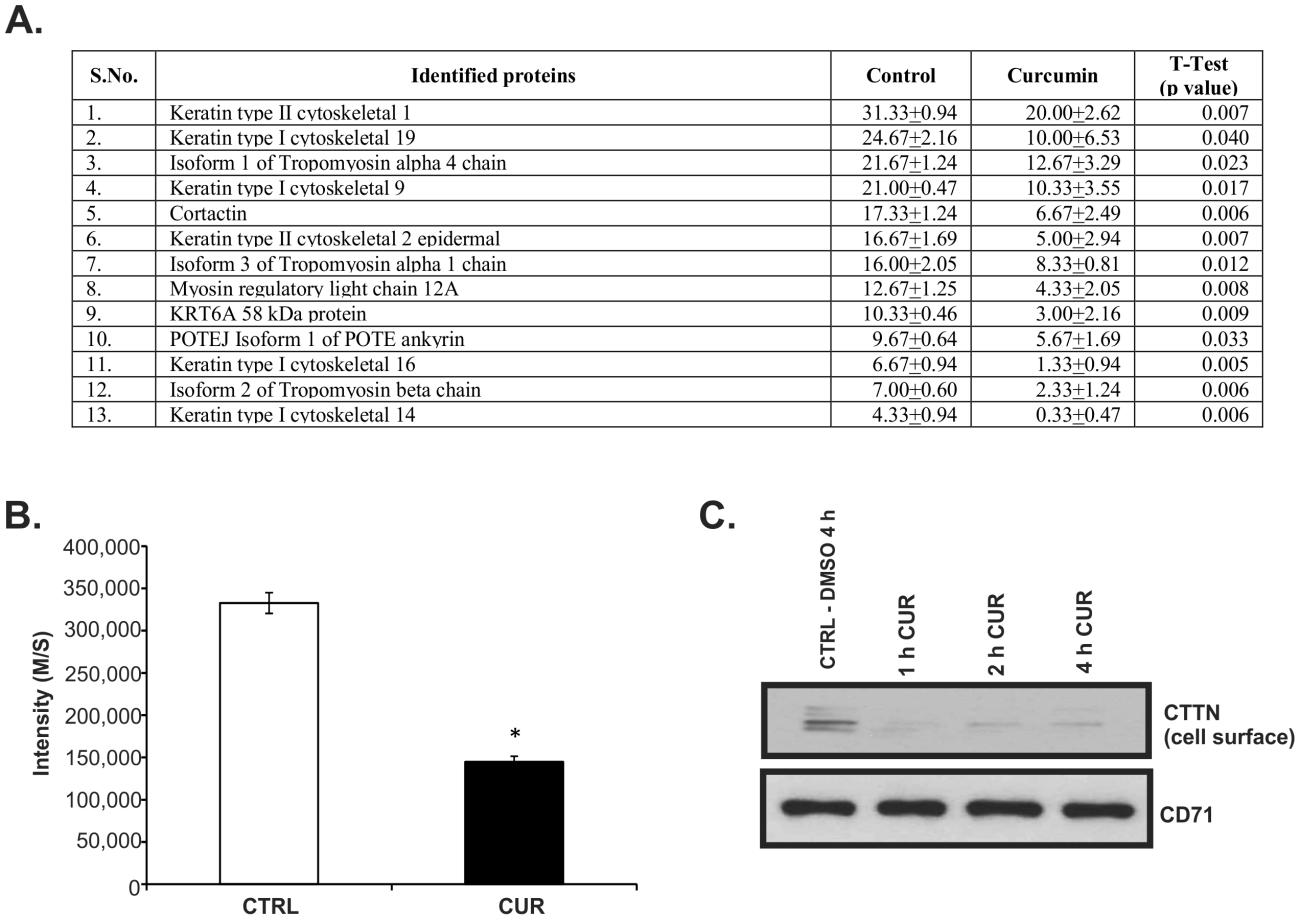
Identification of cortactin as curcumin target in colon cancer cells. (**A**) Table with 13 plasma membrane-associated proteins identified by QTOF-MS/MS as significantly decreased in T84 cell monolayers treated with curcumin. Data shows average number unique peptides identified from three different experiments ± SD. (**B**) Quantitative analysis of cortactin expression in the T84 cells by M/S. The spectrum values were obtained from three different experiments. The quantification data for cortactin protein was derived from M/S using Scaffold proteome software (version Scaffold_3.1.2). Data are means ± SE, *p<0.05 compared with untreated cells, Student's t test. (**C**) Confirmation of CTTN protein expression by western blotting with biotinylated cell surface protein fraction prepared from T84 cells treated with DMSO (CTRL) or 50 µM curcumin for 1–4 hours. CD71 (transferrin receptor) was used as a loading control. CTRL represents cells treated with DMSO for 4 hours.

The T84 colon cancer cells have been extensively used to study the biogenesis of epithelial cell polarity [38] and form well-polarized and tight monolayers when grown on a semi-permeable filter support. We first investigated the distribution and the effects of curcumin on the major membrane-associated proteins in T84 monolayers. To reproduce the exposure to orally-administered (or dietary) curcumin, cells were treated with the compound or DMSO (vehicle) on the apical side. The apical membrane and membrane-associated proteins were then biotinylated, pulled down with streptavidin-conjugated agarose, and analyzed by LC-MS/MS. The results of these experiments are summarized in Fig. 2. 56 proteins were identified by LC-MS/MS. 13/56 showed decreased surface or surface-associated expression ranging from 20–80% in curcumin-treated cells as compared to DMSO control (p<0.0001- p<0.05) (Fig 2A). Only one protein [isoform 1 of leucine rich repeat (in FLII) interacting protein 2, LRRFIP2) showed increased expression (data not shown). Although the function of LRRFIP2 is not completely clear, one report has shown that it plays an important role as an activator of the canonical Wnt signaling pathway, in association with segment polarity protein dishevelled homolog DVL-3, upstream of β-catenin (CTNNB1), leading to the stabilization of the latter [39]. For the purpose of this study, we focused on the decreased levels of cortactin in response to curcumin treatment, because it is frequently overexpressed in cancers and it has been reported to enhance tumor cell migration and invasion [40]. Fig. 2B depicts downregulation of cortactin in the membrane-associated protein fraction from curcumin-treated cells, an observation confirmed by western blotting in cells treated with 50 µM at the apical side for 1–4 hours (Fig. 2C). Transferrin receptor (CD71), used here as a control, showed no change, similar to our previous report [29].

### Dephosphorylation of pTyr^421^-Cortactin by Curcumin

**Figure 3:**
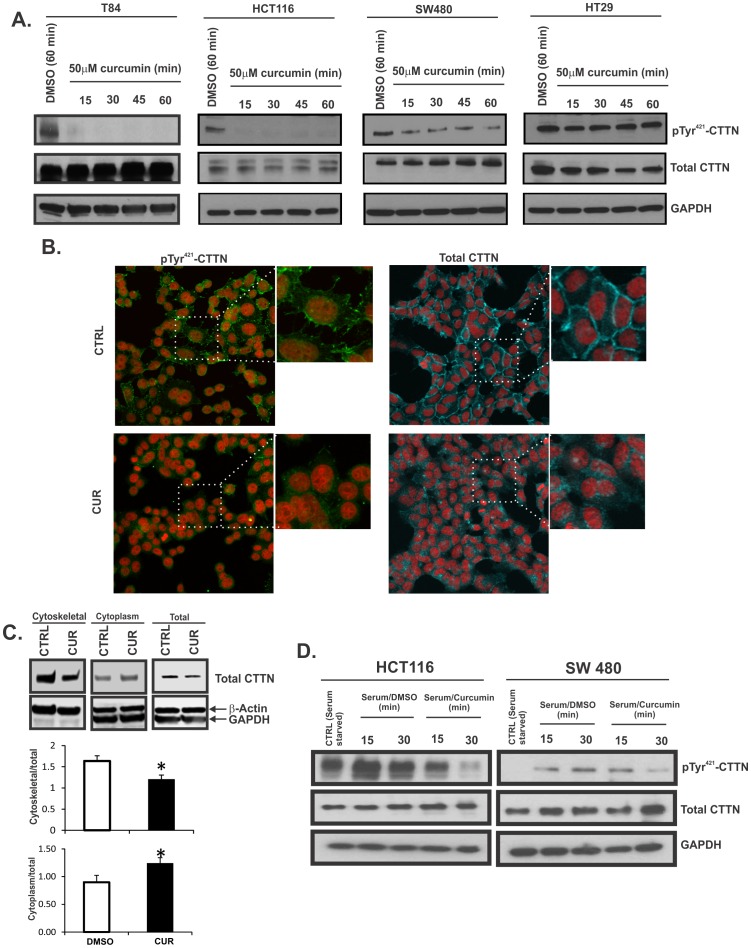
Curcumin induces cortactin dephosphorylation in colon cancer cells. (**A**) T84, HCT116, SW480 and HT29 cells were treated for 15–60 min with DMSO (CTRL) or 50 µM curcumin and pTyr^421^ –CTTN and total CTTN expression was analyzed by western blotting. GAPDH was used as a loading control. (**B**). Immunofluorescent analysis of pTyr^421^ –CTTN (green) and total CTTN (cyan) in HCT116 cells treated with DMSO (top panels) or with 50 µM curcumin for 15 min (bottom panels). Nuclei (red) were counterstained with Sytox Red (Life Technologies). 40 X magnification. Further cropped and magnified images are provided as indicated by the dotted lines. (**C**). Western blot analysis of cortactin, actin and GAPDH proteins from DMSO and curcumin treated cell fractions of HCT116 cells. Total cell lysates were used to represent total protein input. Cytosolic and cytoskeletal proteins were extracted using Cell Fractionation kit (Cell Signaling, MA) and quantification of the blots are summarized in graphs. The images were scanned using C-Digit and quantified using Image Studio Digits (LI-COR Biosciences, NE). The data are expressed as a ratio to total protein (mean ± SD). * p<0.05 DMSO vs. curcumin; Student's T-test. All images are representative of three independent experiments. (**D**). The effects of curcumin in early (15 min) and late (30 min) response of HCT116 and SW480 cells to serum stimulation. The cells were serum-starved overnight and subsequently stimulated with serum-supplemented medium containing DMSO or 50 µM curcumin. pTyr^421^ –CTTN and total CTTN were determined by Western blotting. Representative blots from three experiments are shown. GAPDH served as a loading control. In all cases, CTRL represents DMSO treatment for the longest incubation time in a respective study.

One well-defined mechanism determining aberrant migration of cancer cells is mediated by elevated levels of phosphorylated cortactin, which is triggered by Src and other kinases [41]. Several studies have shown that Src phosphorylates human cortactin mainly at three key tyrosine residues, Tyr^421^, Tyr^470^, and Tyr^486^, resulting in increased cell motility [15]. Cortactin can undergo phosphorylation at these sites upon stimulation with EGF, FGF, PDGF, or after serum starvation. In serum-starved or unstimulated cells, cortactin is normally present in the cytoplasm as a non-phosphorylated protein [42]. Upon growth factor stimulation (EGF, FGF, or PDGF), cortactin is re-distributed from internal cytoplasmic regions to the cortical actin network [42]–[44]. pTyr^421^-Cortactin is enriched in the leading edge of lamellipodia and in podosomes and assists in cell migration. Cortactin tyrosine phosphorylation is progressive, with Tyr^421^ phosphorylation required for subsequent phosphorylation of Tyr^486^ [42]. Since we observed significant increase in the levels of pTyr^421^-cortactin in the majority of the colon tumor samples analyzed, we examined the levels of pTyr^421^-cortactin in colon cancer cell lines following treatment with curcumin. Cortactin phosphorylation at Tyr^421^ was reduced in T84, HCT116, SW480 cells (Fig. 3A) following 50 µM curcumin treatment ranging from 15 min to 1 h. Surprisingly, HT29 cells showed no effect of curcumin on the pTyr^421^-cortactin levels (Fig. 3A). Immunostaining of HCT116 cells treated with curcumin showed a dramatic decrease in pTyr^421^-cortactin signal (Fig. 3B, left panels) accompanied with redistribution of total cortactin away from plasma membrane, and generally weaker signal (Fig. 3B, right panels). The location of cortactin after curcumin treatment in HCT116 cells was also determined by cell fractionation (Fig. 3C). In untreated cells, cortactin was found predominantly in the cytoskeletal fraction, and a small portion was found in the cytosol. Curcumin treatment led to a significant decrease in cortactin levels (p<0.05) in cytoskeletal fraction, with a concomitant increase in cytoplasmic fraction (p<0.05) (Fig. 3C).

In order to determine if curcumin prevents cortactin phosphorylation, we serum-starved HCT116 and SW480 cells followed by stimulation with serum in the presence of DMSO or curcumin. pTyr^421^-cortactin levels increased within 15 min following serum stimulation, however, co-treatment with curcumin did not significantly affect phosphorylation at this early time point (Fig. 3D). However, 30 min exposure to curcumin and serum led to a significant decrease on pTyr^421^-cortactin levels in both cell lines, as compared to cells treated with serum only (Fig. 3D). This observation suggested that curcumin may not act as an inhibitor of cortactin phosphorylation, but that it may induce cortactin dephosphorylation.

### PTPN1 mediates dephosphorylation of pTyr^421^-cortactin in response to curcumin

**Figure 4:**
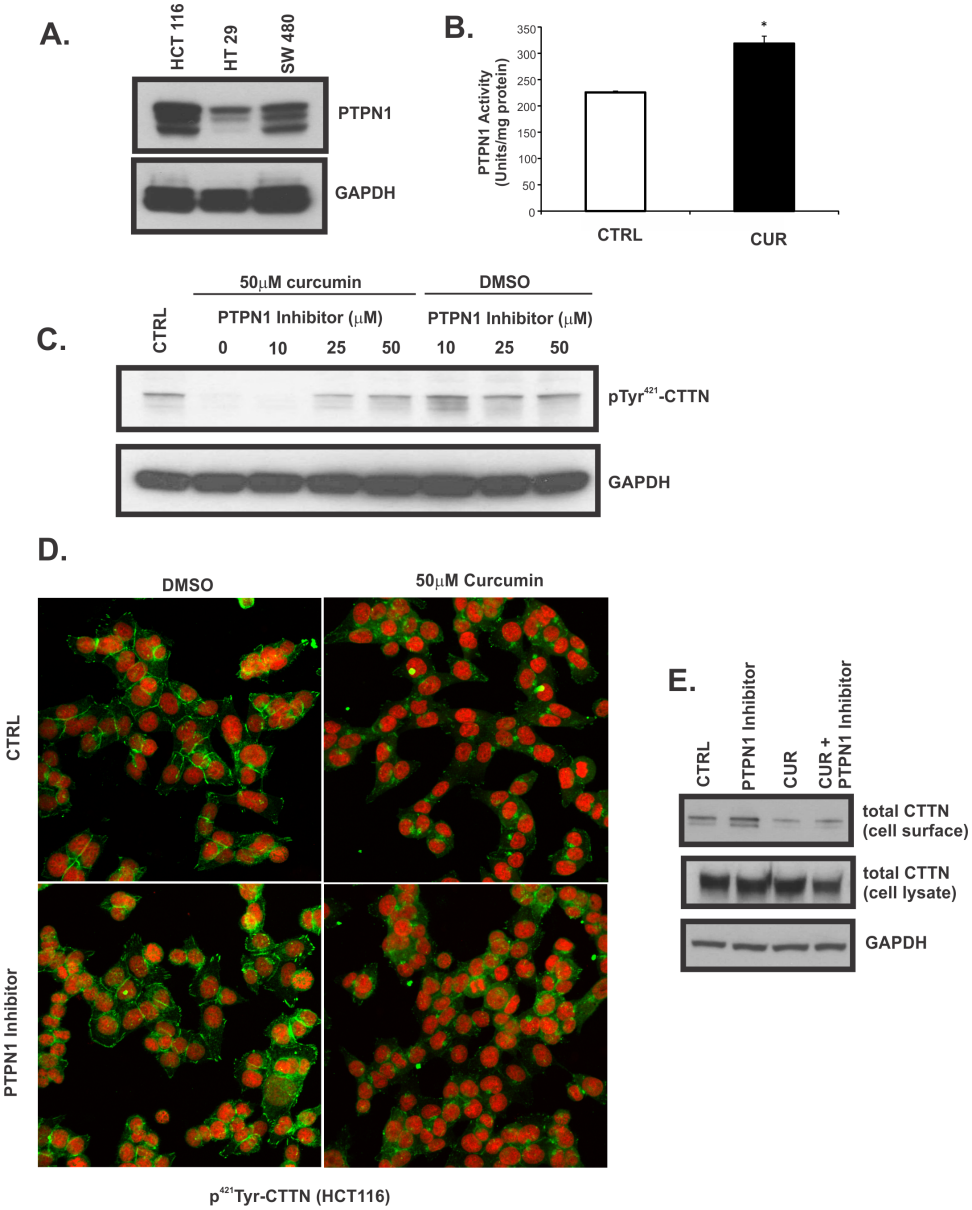
Curcumin dephosphorylates pTyr^421^ –CTTN through activation of PTPN1 in colon cancer cells. (**A**) Expression of PTPN1 protein in HCT116, HT29 and SW480 cells. GAPDH served as a loading control. (**B**) PTPN1 activity in DMSO (CTRL) or curcumin (CUR)-treated HCT116 cells. Equal amounts of protein lysates were assayed for phosphatase activity against DADEpYLIPQQG peptide as substrate as described in Materials and Methods. PTPN1 activity calculating in U/mg protein is shown as means ± SEM from three separate experiments (* p<0.05, CTRL vs. CUR; Student's T-test). (**C**) Dephosphorylation of pTyr^421^-CTTN by curcumin in the presence of PTPN1 inhibitor. Cells were subjected to treatment with 50 µM curcumin or DMSO without or with increasing concentrations of PTPN1 inhibitor for 15 min followed by western blot analysis of pTyr^421^-CTTN. GAPDH served as a loading control. (**D**) Confocal immunofluorescence analysis image of pTyr^421^-CTTN in HCT116 cells treated with DMSO (left panels) or curcumin (right panel) in the absence (top panels) or presence of 50 µM PTPN1 inhibitor. (**E**) Expression of CTTN in HTC116 cells treated as in (D) was evaluated by western blot analysis of cell surface biotinylated fraction and total cell lysate. GAPDH served as a loading control. All images are representative of three independent experiments.

Stuible et al. [45] have shown that PTPN1 phosphatase controls the phosphorylation of cortactin at Tyr^446^ and Tyr^421^. In this study, preincubation of Hela cells with a membrane-permeable PTPN1 inhibitor enhanced hyperosmolarity-induced phosphorylation of these sites and transient overexpression of PTPN1 reduced phosphorylation at both Tyr^421^ and Tyr^446^ [45]. Intriguingly, we observed that expression of PTPN1 in HT29 cells, in which curcumin did not affect the expression of pTyr^421^-cortactin, was dramatically lower than in HCT116 and SW480 cells (Fig. 4A). We tested whether this phenomenon could be explained by mutations or polymorphisms within the PTPN1 coding region, but found no differences among the three cell lines. However, we also analyzed the expression of PTPN1 in human colon cancer biopsies by Western blotting and found no correlation between PTPN1 expression and pTyr^421^-cortactin levels (data not shown). We next sought to determine whether curcumin activates PTPN1 colon cancer cells. Indeed, HCT116 cells treated with 50 µM curcumin had significantly increased activity of PTPN1 when compared to DMSO-treated cells (Fig. 4B). PTPN1 inhibitor XXII abolished the effects of curcumin on pTyr^421^-cortactin expression in a dose-dependent fashion (Fig. 4C). However, immunofluorescence analysis and cell surface biotinylation in HCT119 cells treated with DMSO or curcumin showed that PTPN1 inhibition did not restore the cellular localization of pTyr^421^ or total cortactin, respectively (Fig. 4D, E). These findings suggest that curcumin may trigger dissociation of cortactin from the plasma membrane independently of its effects on Tyr^421^ dephosphorylation.

### Synthesis of Botinylated Curcumin; Physical Binding with PTPN1

**Figure 5:**
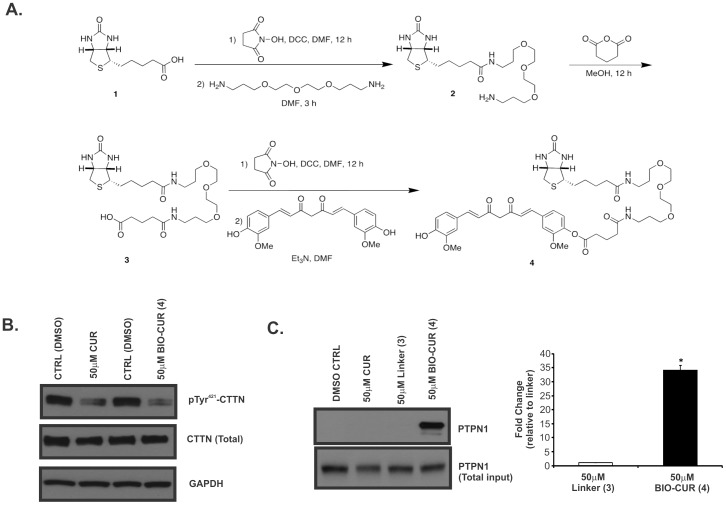
Curcumin physically interacts with PTPN1. (**A**) Synthesis of biotinylated curcumin derivative. (**B**) Comparison of the effects of unmodified curcumin (CUR) and biotinylated curcumin (BIO-CUR) in equimolar concentrations (50 µM) on pTyr^421^-CTTN in HCT116 cells treated for 15 min. GAPDH served as a loading control. (**C**) Western blot analysis (left panel) and quantitative densitometry of the PTPN1 protein from pull-down experiment with biotinylated crcumin. HCT116 cell lysates were prepared with RIPA buffer and combined with curcumin (CUR), biotin linker (compound 3), or biotinylated curcumin (BIO-CUR; compound 4; all at 50 µM) for 30 min at room temperature. Protein fraction recovered with streptavidin agarose beads was analyzed by western blotting for the presence of PTPN1. The data in the summary graph are expressed as fold change compared to linker treated samples from more extended exposures to visualize background PTPN1 signal (mean ± SD). * p<0.05 linker vs. biotinylated curcumin; Student's T-test. All images representative of three independent experiments.

Biotinylated curcumin derivative **4** was synthesized from biotin by modification of a published procedure [35] (Fig. 5A). The known biotinamide **2** was prepared in 85% yield by activation of biotin with *N*-hydroxysuccinamide and DCC in DMF, followed by treatment with 4,7,10-trioxa-1,13-tridecanediamine. The melting point and the ^1^H NMR and ^13^C NMR spectra of **2** were in agreement with the published data [35]. Treatment of biotinamide **2** with glutaric anhydride in methanol gave **3** in 84% yield. Finally, activation of **3** with *N*-hydroxysuccinamide and DCC in DMF, followed by treatment with a five-fold excess of curcumin, gave biotinylated curcumin **4** at 48% yield over the two steps. The ^1^H NMR and ^13^C NMR spectra of **4** were in agreement with the published data [35]. Biotinylated curcumin retained its biological activity as demonstrated by equal inhibition of pTyr^421^-cortactin expression in HCT116 cells treated for 15 min with DMSO (vehicle), or unmodified or biotinylated curcumin in equimolar concentrations (50 µM) (Fig. 5B). To test whether curcumin physically interacts with PTPN1, we performed an in vitro pull down experiment. Pre-cleared HCT116 cell lysate was treated for 30 min at room temperature with equimolar concentrations of unmodified curcumin, biotin linker (compound **3**), or biotinylated curcumin (compound **4**), as depicted in Methods and Fig. 5A, followed by pull-down with streptavidin beads and immunobloting for PTPN1 protein. No PTPN1 was observed in the pulled-down protein fraction from cells treated with DMSO, unmodified curcumin, or biotin linker (Fig. 5C). However, very strong PTPN1 signal was consistently detected from pull-down with biotinylated curcumin (Fig. 5C). The biotinylated curcumin bound proteins were pulled down with Streptavidin-agarose beads and pull down samples were measured by immunoblotting. These results indicate that biotinylated curcumin displays strong and specific physical interaction with PTPN1.

### Colon cancer cell migration is increased by overexpression of cortactin and inhibited by curcumin

**Figure 6:**
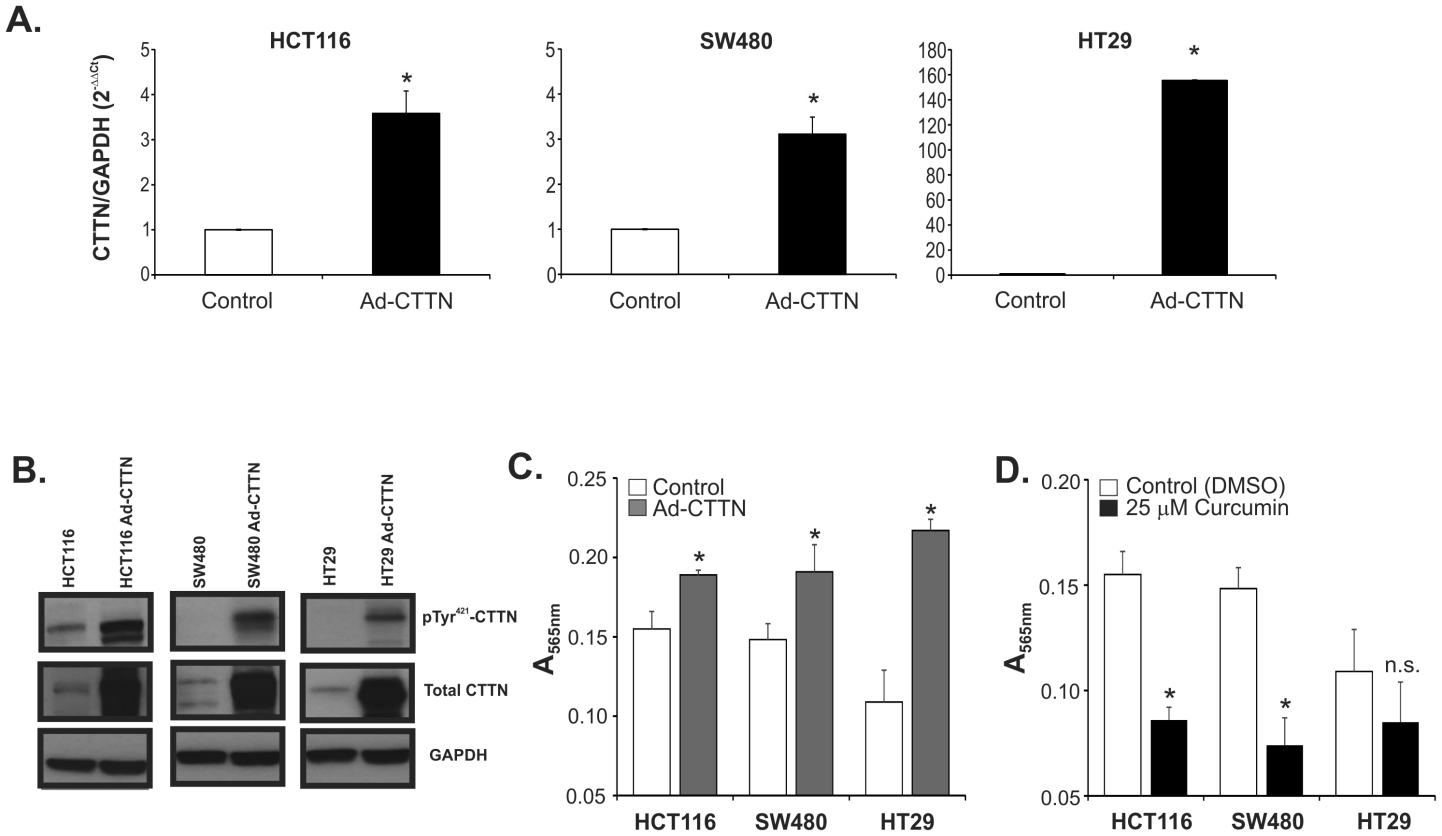
Overexpression of cortactin promotes migration in colon cancer cells; inhibition by curcumin. Ectopic expression of cortactin was accomplished by adenoviral delivery (Ad-CTTN) and elevated expression confirmed by qRT-PCR (**A**) and western blotting (**B**). (**C**) Enhanced migration of HCT116, SW480, and HT29 cells transduced with Ad-CTTN. (**D**) HCT116, and SW480, but not HT29 cells treated with curcumin showed significantly reduced migration. * p<0.05 Control vs. Ad-CTTN or Control vs. Curcumin; Student's T-test. n.s. – not statistically significant. Bars represent mean ± SEM of three independent experiments.

Previous studies have shown that cortactin phosphorylation is important to enhance its function in directing cell migration and invasion by altering the complement of proteins associated with cortactin [8]. Ectopic overexpression of cortactin resulted in increased endothelial and fibroblast cell migration [8], [9]. Since curcumin has been described to reduce the invasion of several types of cancer partly via inhibition of matrix metalloproteases [46]–[49], to avoid the confounding effects of curcumin on proteolitic enzymes, we tested if overexpression of cortactin and/or curcumin can modify HCT116, SW480, and HT29 colon cancer cell migration through uncoated semi-permeable membranes, as described in Materials and Methods. As shown in Fig. 6A, infection of all three colon cancer cell lines with adenovirus encoding CTTN (Ad-CTTN) led to a significant increase in CTTN mRNA expression, which was especially prominent in HCT29 cells. Expression of total CTTN as well as pTyr^421^-CTTN was also significantly elevated (Fig. 6B). All three Ad-CTTN-infected cell lines exhibited significantly increased cell migration in comparison with their respective controls, but consistently with low expression of PTPN1 in HT29 cells, this cell line exhibited a greater response to overexpression of cortactin compared to HCT116 or SW480 (Fig. 6C). Curcumin treatment significantly reduced migration of HCT116 and SW480 cells, but was without statistically significant effect in HT29 cells (Fig. 6D).

### Curcumin reduced interaction of pTyr^421^-CTTN with p120 catenin

**Figure 7:**
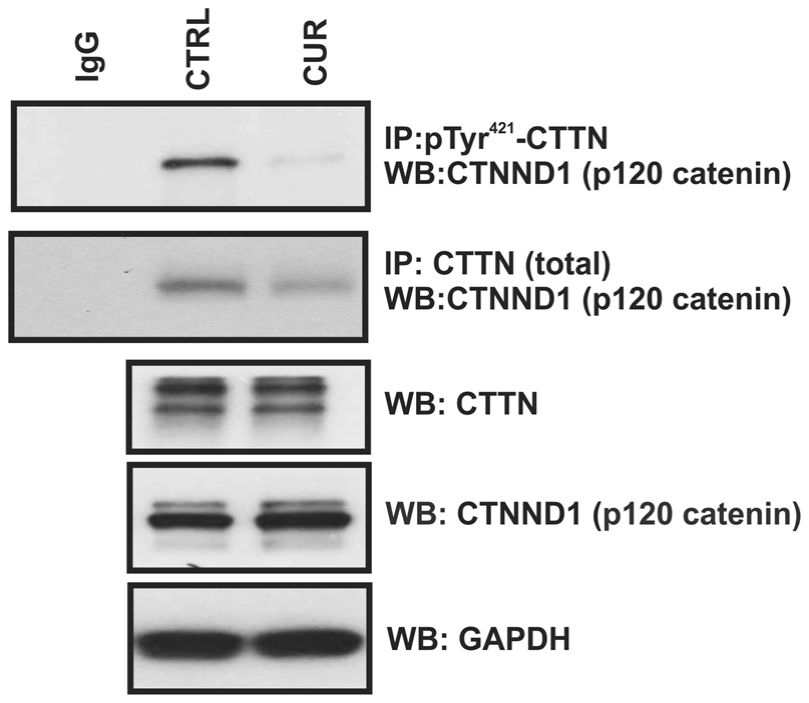
Curcumin impairs the physical interaction between cortactin and p120 catenin (CTNND1). HCT116 cells were treated with 50 µM curcumin for 15 min and pre-cleared lysates were immunoprecipitated using anti- pTyr^421^-CTTN or anti-CTTN (total) rabbit polyclonal antibodies. Immunoprecipitated complexes were analyzed by Western blotting for the presence of CTNND1. Lower three panels demonstrate even input of CTTN, CTNND1 and GAPDH in the cell lysates used for co-immunoprecipitation. All images representative of three independent experiments.

It has been shown that cortactin and p120 catenin (cadherin-associated protein) delta 1 (CTNND1) directly interact with each other via the cortactin N-terminal region to augment the cell migratory ability [50]. CTNND1is colocalized with cortactin-containing actin structures not only at cell–cell junctions, but also at extrajunctional sites, including membrane ruffles and actin-rich halos around endocytic vesicles [50]. We therefore investigated whether curcumin could affect the physical interaction of the proteins by co-immunoprecipitation in HCT119 cells. Curcumin treatment virtually eliminated the interaction of pTyr^421^-CTTN as well greatly reduced the interaction of total CTTN with p120 catenin (Fig. 7). These observations suggest that inhibition of colon cancer cell migration by curcumin is at least in part mediated by disruption CTTN-p120 catenin interaction.

## Discussion

The discoveries which identified the ErbB2 receptor (*HER*2) and epidermal growth factor receptor (EGFR) as target cell surface molecules in breast and colon cancer, respectively, have led to development of effective therapies. Although the toolbox for treating cancer is already quite extensive, most existing drugs inhibit only cancer cell proliferation. The auxiliary treatment aimed at the suppression of cancer metastasis remains an urgent therapeutic need. Prevention of cancer cell dissemination represents an attractive approach as a treatment complementary to conventional chemotherapeutics and biologicals. Based on current knowledge from clinical and basic research studies, cortactin appears to be an attractive target for modulation due to its overexpression in cancer, and its prominent role in cell motility. In our study, western blot and immunohistochemical analysis showed that pTy^421^-cortactin was overexpressed in nearly 70% of the tumors examined, suggesting that pTy^421^-cortactin levels could be a potential biomarker for colorectal cancer. The studies of breast, head/neck squamous cell cancer, and renal cancers have shown that increased cortactin expression resulted from gene amplification of chromosome 11q13. Cortactin overexpression could be associated with poor survival and aggressive behavior of metastatic cancers [51]–[53]. Previously, it was shown that Src kinase can bind with cortactin protein [14], [44], [54] and phosphorylates three tyrosine residues (Tyr^421^, Tyr^466^ and Tyr^482^) located within cortactin's proline-rich domain [8]. Expression of cortactin with mutations at these residues significantly reduced cortactin tyrosine phosphorylation [8]. Previously, Head et al., [42] have shown that the presence of a pTyr^421^ is required for phosphorylation of Tyr^466^ in murine cortactin in vivo, but that phosphorylation of Tyr^421^ and Tyr^466^ is independent of Tyr^482^ phosphorylation. Our study reveals that the mechanisms of overexpression of total and pTyr^421^-cortactin in human colon cancer is different from that reported in other cancers and results not from gene amplification, but rather from post-translational modifications, resulting in enhanced phosphorylation and likely increased protein stability. Moreover, overexpression of cortactin using adenoviral delivery in three colon cancer cell lines promoted cell migration, thus highlighting the role of cortactin in colon cancer cell motility.

Curcumin, a small bioactive molecule derived from a common spice (Turmeric) has been considered as potentially very effective and well-tolerated adjuvant therapy in cancer and inflammation [55], [56]. Among other mechanisms of action, cell cycle arrest, induction of apoptosis, regulation of epigenetic events, inhibition of angiogenesis, chemo- and radio-sensitization, have resulted in many preclinical studies and clinical trials. Curcumin's ability to inhibit matrix metalloproteinases, cell migration and invasion [47], [57]–[60] also suggests its potential utility as supplemental treatment to reduce cancer metastasis. In this report, we also provide the first evidence that curcumin induces cellular redistribution of cortactin away from the plasma membrane, induces cortactin dephosphorylation through a direct physical interaction with PTPN1 protein tyrosine phosphatase to increase its activity, and to reduce cancer cell migration. Protein-tyrosine phosphatase 1B (PTPN1) has been reported to bind cortactin at the Tyr^421^ and Tyr^446^ phosphorylation sites to induce dephosphorylation [45]. Several lines of evidence support our conclusions about the role of PTPN1/cortactin axis in the mechanism of curcumin-induced inhibition of colon cancer cell migration. Curcumin was ineffective in inhibiting the initial activation of cortactin (Tyr^421^ phosphorypation) in response to serum stimulation, thus suggesting dephosphorylation as a more likely mechanism. Inhibition of PTPN1 abolished curcumin-induced dephosphorylation of pTyr^421^-cortactin whereas curcumin treatment increases PTPN1 activity. Moreover, we synthesized a biotinylated derivative of curcumin, demonstrated preserved biological activity, and showed that curcumin physically interacts with PTPN1. Since curcumin increases PTPN1 activity, while PTPN1 inhibitor XXII prevents curcumin-induced cortactin dephosphorylation (Fig. 4C), it is plausible that a curcumin binding site within the CTTN protein is the same or is in close proximity to that of PTPN1 inhibitor.

Interestingly, curcumin was significantly less effective in reducing the levels of pTyr^421^-cortactin in HT29 cells, which had dramatically lower expression of PTPN1 compared to HCT116 and SW480 cells. The basis for the difference in PTPN1 expression among these cell lines is not clear, although we excluded potential mutations within the PTPN1 coding region. Consistent with low expression of PTPN1, HT29 cells responded to overexpression of cortactin with a much greater increase in their migration. Consistently, curcumin did not significantly inhibit HT29 cell migration, as compared to the other two cell lines tested. However, in the tested tumor samples, PTPN1 expression was not affected, thus raising a possibility that relative PTPN1 deficiency should not be a factor limiting the efficacy of curcumin or other drugs targeting PTPN1/cortactin in potentially reducing colon cancer cell migration and metastasis.

Several reports have indicated that p120 catenin (CTNND1), which belongs to the catenin family of cell adhesion proteins [61], can associate with cortactin, an interaction important in regulating cell motility [62], [63]. We found that in colon cancer cells, cortactin interacts with CTNND1, and that curcumin strongly decreased this association, likely due to diminished cortactin phosphorylation at Tyr^421^. We were not able to confirm these findings by co-localization experiments using immunofluorescence due to the strong immunostaining with p120 antibody within the cytoplasm (not shown). Hence, we postulated that disruption of CTNND1-cortactin interaction may be secondary to curcumin-induced dephosphorylation of cortactin by PTPN1 and may contribute to its effects of curcumin on cancer cell migration.

In conclusion, we have shown that pTyr^421^-cortactin is overexpressed in colon cancer, that curcumin modulates the activity of PTPN1 phosphatase to decrease cortactin phosphorylation and interaction with CTNND1, and ultimately to decrease colon cancer cell migration. To further develop and implement colon cancer chemoprevention strategies, additional studies are needed to determine the mechanism by which curcumin activates PTPN1, as well as defining a broader role that PTPN1/cortactin play in cancer cell metastasis.
